# Effects of Novel Dental Composites on *Streptococcus mutans* Biofilms

**DOI:** 10.3390/jfb15010013

**Published:** 2023-12-29

**Authors:** Rayan B. Yaghmoor, Mohammad Abdel-Hadi, Haralampos Petridis, Elaine Allan, Anne M. Young

**Affiliations:** 1 Department of Restorative Dentistry, College of Dental Medicine, Umm Al-Qura University, Makkah 24381, Saudi Arabia; rbyaghmoor@uqu.edu.sa; 2 Unit of Prosthodontics, Department of Restorative Dentistry, UCL Eastman Dental Institute, Rockefeller Building, London WC1E 6HX, UK; mohammad.hadi87@gmail.com (M.A.-H.); c.petridis@ucl.ac.uk (H.P.); 3 Department of Microbial Diseases, UCL Eastman Dental Institute, Royal Free Hospital, London NW3 2QG, UK; e.allan@ucl.ac.uk; 4 Department of Biomaterials and Tissue Engineering, UCL Eastman Dental Institute, Royal Free Hospital, London NW3 2QG, UK

**Keywords:** dental composite, *Streptococcus mutans*, biofilm, antibacterial, biomass, exo-polysaccharide, extracellular DNA

## Abstract

With the phase-out of amalgam and the increase in minimally invasive dentistry, there is a growing need for high-strength composite materials that can kill residual bacteria and promote tooth remineralization. This study quantifies how antibacterial polylysine (PLS) and re-mineralizing monocalcium phosphate monohydrate (MCPM) affect *Streptococcus mutans* biofilms and the strength of dental composites. For antibacterial studies, the MCPM-PLS filler percentages were 0-0, 8-4, 12-6, and 16-8 wt% of the composite filler phase. Composite discs were immersed in 0.1% sucrose-supplemented broth containing *Streptococcus mutans* (UA159) and incubated in an anaerobic chamber for 48 h. Surface biomass was determined by crystal violet (CV) staining. Growth medium pH was measured at 24 and 48 h. Biofilm bacterial viability (CFU), exo-polysaccharide (water-soluble glucan (WSG) and water-insoluble glucan (WIG)), and extracellular DNA (eDNA) were quantified. This was by serial dilution plate counting, phenol-sulfuric acid microassay, and fluorometry, respectively. The biaxial flexural strengths were determined after water immersion for 1 week, 1 month, and 1 year. The MCPM-PLS wt% were 8-4, 8-8, 16-4 and 16-8. The normalized biomass, WSG, and WIG showed a linear decline of 66%, 64%, and 55%, respectively, as the PLS level increased up to 8%. The surrounding media pH (4.6) was all similar. A decrease in bacterial numbers with the 12-6 formula and a significant reduction with 16-8 compared to the 0-0 formulation was observed. The eDNA concentrations in biofilms formed on 12-6 and 16-8 formulations were significantly less than the 0-0 control and 8-4 formulations. Doubling MCPM and PLS caused a 14 and 19% reduction in strength in 1 week, respectively. Average results were lower at 1 month and 1 year but affected less upon doubling MCPM and PLS levels. Moreover, a 4% PLS may help to reduce total biomass and glucan levels in biofilms on the above composites. Higher levels are required to reduce eDNA and provide bactericidal action, but these can decrease early strength.

## 1. Introduction

Dental caries is the most common disease worldwide with approximately 2 billion and 0.5 billion people suffering from decay in permanent and primary teeth, respectively [1]. Untreated decay can cause pain and lead to more serious abscesses and sepsis. With the phase-out of amalgam fillings, the dental composite is the major alternative. The composite, however, is more expensive and time-consuming to place in addition to having higher failure rates [2]. The most common cause of dental composites’ failure is recurrent caries [3,4,5]. This is mainly because of their greater tendency to accumulate dental biofilms and bacterial penetration in gaps between the tooth and the restoration [6]. Furthermore, with an increasing drive to more minimally invasive dentistry, there is a greater risk of residual bacteria beneath restorations.

In order to address the above issues, many [7] dental composites that release antibacterial agents have been developed. A major difficulty, however, is ensuring the antibacterial agent can be released from the composite at sufficient rates to affect the high bacterial concentrations that may be present in a minimally excavated carious tooth. Furthermore, much higher concentrations of antibacterial agents are generally required to affect bacteria protected within the biofilms that form when cariogenic bacteria and sucrose are present. This must be achieved without causing increased cytotoxicity to pulp cells. Additionally, formulations that promote re-mineralization of the affected tooth structures, through the release of fluoride, calcium, or phosphate ions, have been produced [4,5]. Of concern with both antibacterial and re-mineralizing composites, however, is increased fracture risk due to reduction in strength upon water sorption and component release.

Previously, formulations that contain monocalcium phosphate monohydrate (MCPM) and polylysine (PLS) were described [4,5]. Both are extensively used in food and are generally recognized as safe status. During composite paste placement, these two hydrophilic components may absorb water from the tooth surface. This, in combination with an adhesion-promoting monomer, enables the hydrophobic composite to adapt better to hydrophilic tooth surfaces. It also enables effective sealing of hand-excavated tooth structures and halts enzyme-catalyzed hydrolysis of demineralized dentine [4]. Following composite polymerization, MCPM and PLS encourage water sorption-induced swelling to compensate for polymerization shrinkage. Additionally, MCPM provides a self-repair mechanism through precipitation of apatite that occupies a greater volume. This may seal gaps at the restoration/tooth interface. It also fills cracks in the material if fractured, or holes when a component is released [4]. The ability of MCPM to promote re-mineralization at a demineralized dentine interface has been demonstrated using simulated body fluid [4]. Together these features may help to prevent longer-term bacterial microleakage and recurrent caries. Additionally, the above materials provide early release of PLS which may kill residual bacteria and reduce biofilm development under a restoration.

Understanding biofilm structure development on the above materials is beneficial to identify what is a suitable level of composite PLS to reduce their growth. Biofilms consist of an accumulation of bacterial cells (microcolonies) attached to surfaces and surrounded by a self-secreted extracellular matrix (ECM) (≈50–90% of the biofilm weight). The ECM provides physical integrity, mechanical stability, and bulk to the biofilm matrix and aids antimicrobial resistance [8]. The ECM network contains exo-polysaccharides, proteins, and extracellular DNA (eDNA) [9]. The exo-polysaccharides are mainly composed of water-soluble (WSG) and water-insoluble (WIG) glucans. Bacterial microcolonies occupy a significant part and initiate and propagate their formation [8].

One of the main bacterial contributors in cariogenic biofilms is Gram-positive *Streptococcus mutans* of which the serotype c UA159 strain was the first to be genome sequenced [10,11]. With time, increasing acidity can make the biofilm suitable only for acid-tolerant and lactate-consuming cocci species (ecological plaque hypothesis) [10]. The main by-product of *S. mutans* upon fermentation of sucrose and other sugars is lactic acid. The low-pH environment is the direct cause of tooth demineralization and caries initiation and progression [10]. As a result, aciduric species (e.g., *S. mutans*) dominate the dental plaque microbiota [12]. Both a reduction in species richness and an increase in the number of *S. mutans* have been correlated with caries progression [13].

Previous work demonstrated a linear reduction in surface *S*. *mutans* biofilm mass and thickness with increasing composite PLS content [14]. With increasing PLS, there was an abrupt change from primarily live to largely dead bacteria within the biofilm. Therefore, this work aimed to gain a better understanding of how polylysine in composites affects bacterial viability and surface biofilm composition. In the earlier work [14], an aerobic environment was employed for biofilm growth. This would be expected at the margins of the restoration where bacterial microleakage will initiate. An anaerobic environment is arguably, however, a better model for beneath a restoration, where early antibacterial action is important if there are residual bacteria. The present study will therefore assess if a similar reduction in biofilm mass occurs with biofilms produced on the same composites but in an anaerobic environment. Additionally, new studies include measuring the pH of the surrounding media, viable bacterial counting, and quantifying exo-polysaccharide (WSG and WIG) and eDNA. Furthermore, mechanical properties are described to demonstrate whether increasing the hydrophilic MCPM and PLS components has a significant detrimental effect. The null hypotheses are that varying composite PLS and MCPM levels have no significant effect on these properties and time has no significant effect on mechanical strength.

## 2. Materials and Methods

### 2.1. Composite Pastes and Discs Preparation

Composite components/sources are described in detail in a previous publication [14]. The liquid phase was of fixed composition. It was mixed by adding 4-methacryloyloxyethy trimellitate anhydride (4-META, 3 wt%) and camphorquinone (CQ, 1 wt%) to poly (propylene glycol) dimethacrylate (PPGDMA, 24 wt%). This was stirred for two hours at room temperature (RT) until a clear liquid was obtained. Urethane dimethacrylate (UDMA, 72 wt%) was then added and stirred for 24 h until the liquid was again clear. This phase was mixed in a brown (blue light-proof) glass container using a magnetic stir bar on a magnetic multichannel stirrer hot plate (Jeio Tech MS-52M, Daejeon, Republic of Korea). No additional inhibitors were added to the monomers as there was sufficient included by the manufacturers to ensure long-term paste stability. This was assessed using accelerated high-temperature aging and long-term aging at 4 °C with FTIR.

The composite filler phase contained two different sizes of silane-treated, radiopaque strontium aluminosilicate glass particles (7 μm: 0.7 μm 2:1). Non-silane-treated nano silica (40 nm, 10 wt%) was added. PLS (Handary, Brussels, Belgium) was added to this powder at 0, 4, 6, or 8 wt%. Monocalcium phosphate monohydrate (MCPM) (Himed, Old Bethpage, NY, USA) was added at 0, 8, 12, or 16 wt%.

The powder was mixed for 10 s in a Speedmixer (DAC600.2 CM51, Synergy Devices Ltd., High Wycombe, UK) at 3500 rpm to evenly distribute different powder particles. The liquid phase was then added to the powder phase at a powder-liquid ratio of 3:1 (wt/wt) and mixed for 40 s in the Speedmixer at the same speed. Resultant pastes were stored at room temperature in opaque (light-proof) containers until used.

For antibacterial studies, four composites were formulated. These are given the codes 0-0, 8-4, 12-6, and 16-8. These indicate the wt% of MCPM-PLS, respectively, in the filler. The formulations selected enable trends to be assessed versus increasing PLS whilst maintaining a constant MCPM/PLS weight ratio of 2. For mechanical tests, four formulations containing MCPM-PLS of 8-4, 8-8, 16-4, and 16-8 were produced. These compositions were based on a 2-level, 2-variable factorial design which enables separation of the effects of MCPM versus PLS. The 8-4 formula has been full-scale manufactured by Schottlander Dental Company (Letchworth, UK) (tradename Renewal MI) for clinical trials.

Discs (9.4 mm diameter, 1 mm thick, and 0.13 g) were prepared by placing the pastes within a metal circlip mold and pressing it between two clear acetate sheets before light-curing. Each surface (top and bottom) was exposed to four overlapping irradiation cycles (40 s each) to ensure maximum cure [15]. The curing unit directly contacted the sheets. It was a light-emitting diode (LED) with a 450–470 nm wavelength and power of 1100–1300 mW/cm^2^ (Demi Plus, Kerr Dental, Bioggio, Switzerland) with a diameter tip of 8 mm. After curing, composite discs were removed from the molds, and any excess composite trimmed and smoothed with fine grit-paper (size 1200). Discs were sterilized by UV light for 40 min on the top and the bottom surfaces before use. Additionally, copper discs (copper foil, Sigma–Aldrich, Gillingham, UK) with the same surface area were used as a positive control surface as required by ISO 3990 [3].

### 2.2. Biofilm Formation

Anaerobic biofilms were produced as described in ISO 3990 Section 7.3.2 directly on the set composite and copper surfaces [3]. Biofilms formed on 3 separate days were used for biofilm mass quantification. Those produced on a further 3 days were used for selective components analysis. For each material, 3 samples were employed at each time point.

For biofilm formation, composite/copper discs were placed in the bottom of sterile 24-well plates. 1 mL brain heart infusion (BHI) broth supplemented with 0.1% (*w*/*v*) sucrose was added to each well. *S. mutans* (UA159), obtained from an overnight (16 h) culture, was included at a concentration of 1 × 10^6^ colony-forming units per milliliter (CFU/mL). The well plate was incubated at 37 °C for 48 h in an anaerobic chamber (Whitley A95, Don Whitley Scientific, Bingley, UK). The medium was renewed after the first 24 h. Three wells with no disc were used as negative controls to detect plate contamination; these contained broth and sucrose only. After 48 h, the medium was removed, and the biofilms were washed twice with 1 mL of sterile phosphate-buffered saline (PBS).

### 2.3. Relative Biofilm Mass

Crystal violet (CV) staining was used to quantify relative biofilm mass on the 4 composites and copper control [14,16], three independent repetitions with three discs per composite formulation per repeat (n = 3) were included. Briefly, to fix the biofilms, discs were transferred to new 24 well plates and 1 mL of methanol (AnalaR NORMAPUR, Avantor, VWR, Lutterworth, UK) was added. After 15 min, the methanol was replaced by 1 mL of aqueous 0.1% CV. Non-absorbed CV was removed after 5 min, through washing with 1 mL of PBS twice. Following drying at 50 °C, bound CV was dissolved using 1 mL of 30% acetic acid. The resultant solutions were diluted to enable the determination of absorbance at 595 nm (spectrophotometer, Biochrom WPA CO8000, Cambridge, UK). Results are reported as absorbance multiplied by the dilution.

### 2.4. Evaluation of Biofilms Selective Components

To identify how altering composites’ PLS and MCPM levels affect various biofilm components, in separate experiments from above, the pH of the surrounding media at 24 and 48 h was determined. Additionally, biofilms were resuspended in a universal tube containing 2 mL of PBS and 0.05 g glass beads (180 μm) (Sigma–Aldrich, Taufkirchen, Germany), then vortexed and mixed for 15 s. Portions were collected for viable bacteria, WSG, WIG, and eDNA quantification. WSG and WIG quantification was undertaken for the 0-0, 8-4, and 16-8 formulations only. Three independent repetitions with three discs per composite formulation per repeat (n = 3) were included. All four composite formulations and the copper control were used in pH, viable bacteria, and eDNA studies described below.

### 2.5. PH Measurements

To evaluate growth media acidity at 24 and 48 h, a digital pH meter (pH meter 240, Corning, NY, USA) was employed. Commercial buffer solutions at pH 4, 7, and 10 were used for meter calibration.

### 2.6. Viable Bacteria Counting

To determine viable bacterial counts, 20 mL of each resuspended biofilm was diluted by multiple factors of 10 to provide a dilution series. 100 μL aliquots of each dilution were spread on BHI agar plates (3 per dilution). After 72 h of incubation in air enriched with 5% CO_2_, the number of bacterial colonies on each plate was counted. The results are reported as CFU/mL of the resuspended biofilm.

### 2.7. WSG and WIG Quantification

For WSG and WIG determination, the resuspended biofilms were centrifuged (4000 rpm for 15 min). The supernatant containing the WSG and eDNA was filtered (Millipore Express 0.22 μm, Darmstadt, Germany). WIG, in the sediment, was dissolved in 2 mL of 0.4 M NaOH and separated from insoluble material by centrifugation at 4000 rpm for 15 min. Following filtration (Millipore Express, 0.22 μm), WIG solutions were dialyzed in water using Slide-A-MINI Dialysis Devices (ThermoFisher Scientific, Loughborough, UK) with a 10 kDa molecular weight cutoff (MWCO) membrane to remove NaOH and prevent interference with the phenol sulfuric acid assay.

WSG and WIG concentrations were determined as in previous studies using a phenol-sulfuric acid reagent [17,18]. To concentrate the WSG and WIG samples, portions were freeze-dried (Hero drywinner/Edwards RV5 rotary vane pump, Becker, UK) and resuspended in 120 μL of deionized water. In total, 30 μL of the resuspension was placed in a microtiter plate 96-well with 6.5% phenol (30 μL) and absolute sulfuric acid (150 μL). After pipette-mixing three times, the plate was covered with foil to prevent light exposure and then incubated at RT for 30 min. Resultant absorbance at 490 nm (A_490_) was measured using a spectrophotometer microplate reader (SPECTROstar^Nano^, BMG LABTECH, Ortenberg, Germany). A calibration curve, generated using glucose concentrations of 0 (blank), 4, 8, 12, 16, and 20 μg/well, was used to convert A_490_ into WSG and WIG μg/mL of the resuspended biofilm.

### 2.8. eDNA Quantification

For eDNA determination, 400 μL recovered from the supernatant in the above section was mixed with 40 μL of 3 M Na-acetate (pH 5.2) and 800 μL absolute ethanol. This was stored at −20 °C overnight to precipitate the DNA. The DNA was collected by centrifuging (microcentrifuge 5424, ThermoFisher Scientific, Loughborough, UK) at 15,000 rpm for 10 min. Following washing with 0.5 mL of 70% ethanol, eDNA was again separated by centrifugation and dried at RT for 1 h. Once dry, the DNA was redissolved in 150 mL of water. Its concentration was determined using a QubitTM dsDNA HS (double-strand DNA high sensitivity) assay kit (Invitrogen) and a Qubit fluorometer (QubitTM 3 fluorometer, Invitrogen, ThermoFisher Scientific, Loughborough, UK) (accuracy range from 0.5 to 500 ng/mL).

### 2.9. Biaxial Flexural Strength Determination

For biaxial flexural strength determination, a total of 72 discs of the four formulations 16-8, 16-4, 8-8, and 8-4 were prepared as above. Next, 24 h after light cure, 18 discs of each formulation were equally divided into 3 sterile tubes each containing 10 mL of deionized water. The 3 tubes were incubated for 1 week, 1 month, or 1 year at 37 °C prior to flexural strength determination (n = 6 per time point and material). Strength was determined using a Shimadzu Autograph (AGS-X, Kyoto, Japan) with a crosshead speed of 1 mm/min and ball on ring jig. The ball diameter was 4 mm, and the sample was placed directly on the 8 mm diameter metal ring support. Flexural strength, S, was calculated using:

$$S=\frac{F}{l^{2}}\left\{\left(1+\nu \right)\left[0.485ln\left(\frac{r}{l}\right)+0.52\right]+0.48\right\}$$

F is the load at failure, l is the specimen’s thickness, r is the radius of a circular support (8 mm) and ν is Poison ’s ratio (0.3) [5].

### 2.10. Statistical Analysis and Equation Fitting

Data were analyzed by Statistical Package for the Social Sciences (SPSS 25.0 for Mac, IBM, Chicago, IL, USA) software. The significance level was 0.05 (*p* < 0.05). For antibacterial studies one-way Analysis of Variance (ANOVA) was utilized, followed by the post-hoc Tukey’s HSD test for multiple comparisons. The 95% confidence intervals (CI) provided primarily indicate the biofilm variability on different days. To reduce the effect of this variability, normalized biofilm mass, WSG, and WIG for the experimental materials were also obtained by dividing results on a given day by values for the 0-0 formulation. Line fitting to this normalized data and R^2^ calculations (Pearson product–moment correlation function) were undertaken using Excel.

For factorial analysis of strength data, the following equations were used with Microsoft Excel version 16.79.1 [19]:


$$lnS=<lnS>\pm a_{1}\pm a_{2}\pm a_{1,2}$$

$$D(\%)=100\left(\frac{1}{{\exp(2a}_{i})}-1\right)$$

a_1_, a_2,_ and a_1,2_ quantify the level of effect of the MCPM, PLS, and any interaction between the two variables, respectively. D is the percentage decline in strength due to variable, i, or an interaction effect. Moreover, 95% CI for these parameters was calculated by fitting the above equations to 6 sets of data at each time point. Each data set had one result from each of the 4 different formulations. Effects of MCPM and PLS were considered significant if 95% CI values and interaction terms were both smaller than the effect of the variable [19]. Additionally, the Kruskal–Wallis one-way analysis of variance (ANOVA) (*p* < 0.05) was used to assess if time had significant effects.

## 3. Results

### 3.1. Biofilm Mass

**Figure 1:**
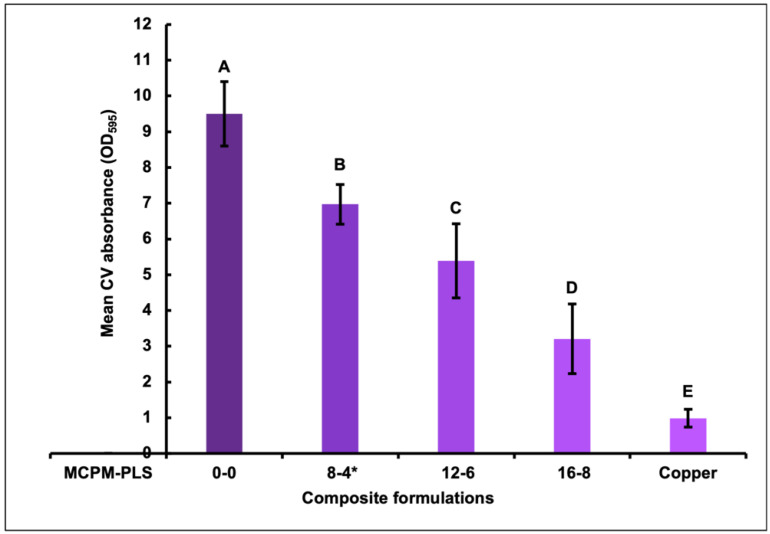
Mean crystal violet (CV) absorbance ± (95%CI, n = 3) absorbed by biofilms on the four composite formulations (0-0, 8-4, 12-6, and 16-8) and copper discs (error bars are for three independent repetitions with an average result of three discs per material per repeat (n = 3)). Materials with the same uppercase letter/s above the bars were not significantly different at *p* < 0.05. * Renewal MI formulation. MCPM-PLS numbers indicate their weight percentage in the powder.

The CV quantification indicated a decline in biofilm mass upon increasing PLS concentration in the composite formulations. All the PLS-containing formulations and the copper control had significantly less absorbed CV than the 0-0 composite (with no PLS); 26%, 44%, 66%, and 90% for 8-4, 12-6, 16-8, and copper, respectively. Additionally, the 16-8 formula (with 8 wt% PLS in the filler) showed significantly less biomass than the other two PLS-containing formulations, by 54% and 41% for 8-4 and 12-6, respectively. Furthermore, the 12-6 formula (with 6 wt% PLS) had significantly less biomass than the 8-4 formula (with 4 wt% PLS), by 23%. However, all composites had significantly more biomass than copper (Figure 1).

### 3.2. Biofilm Acidogenesis (PH)

**Figure 2:**
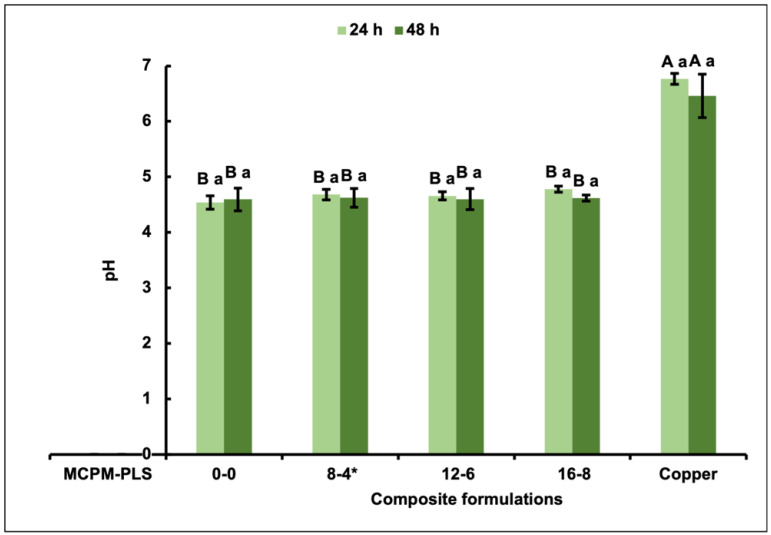
Mean pH (24 and 48 h) ± (95%CI, n = 3) of media surrounding biofilms formed on the four composite formulations (0-0, 8-4, 12-6, and 16-8) and copper discs (error bars are for three independent repetitions with an average result of three discs per material per repeat (n = 3)). Materials at the same time point, with the same uppercase letter/s above the bars were not significantly different (*p* < 0.05). For a given material, the same lowercase letter above the bars indicates no significant difference (*p* < 0.05) between the 2 time points * Renewal MI formulation. MCPM-PLS numbers indicate their weight percentage in the powder.

The pH data showed that all biofilms formed on the composite formulations have the same acidogenesis effect on the surrounding media regardless of measurement time. Additionally, biofilms on all formulations showed significantly more acidogenicity than the biofilms formed on the copper (Figure 2).

### 3.3. Bacterial Viability Counting

**Figure 3:**
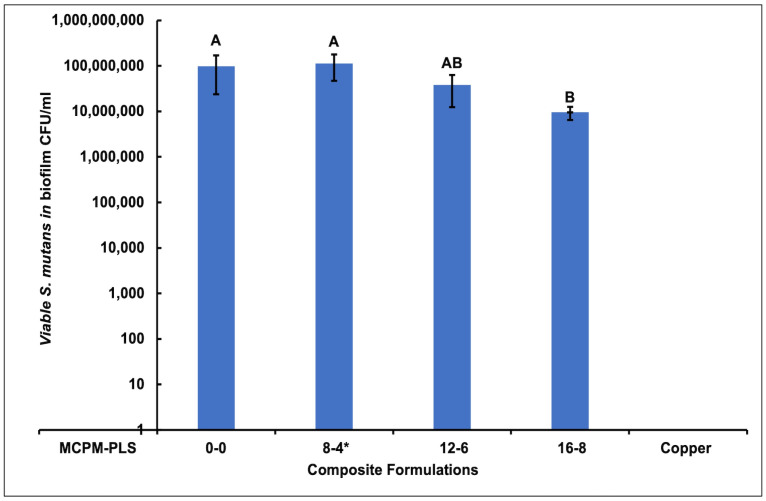
Mean viable *Streptococcus mutans* ± (95%CI, n = 3) (CFU/mL) in biofilms formed on the four composite formulations (0-0, 8-4, 12-6, and 16-8) and copper discs (error bars are for three independent repetitions with an average result of three discs per material per repeat (n = 3)). Material with the same uppercase letter/s above the bars was not significantly different at *p* < 0.05. * Renewal MI formulation. MCPM-PLS numbers indicate their weight percentage in the powder.

The viable counting revealed no significant difference in the number of *S. mutans* between biofilms formed on the composite formulations except for the 16-8 formula, which showed significantly less viable bacteria than the 0-0 and 8-4 formulations 90% and 92%, respectively. No viable bacteria were detected in biofilms formed on the copper discs (Figure 3).

### 3.4. Exo-Polysaccharide Quantification (WSG/WIG)

**Figure 4:**
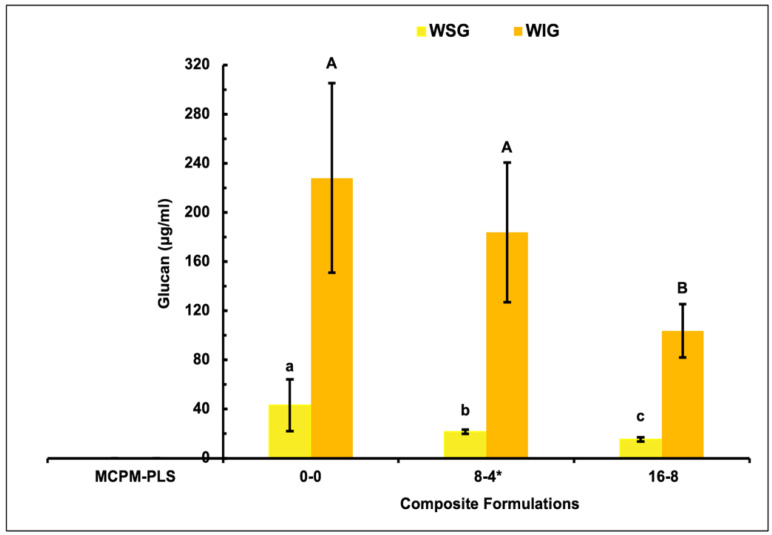
Mean water-soluble (WSG) and water-insoluble (WIG) glucan ± (95%CI, n = 3) (μg/mL) of biofilms formed on the three composite formulations (0-0, 8-4, and 16-8) (error bars are for three independent repetitions with an average result of three discs per material per repeat (n = 3)). Composite formulations, with the same uppercase/lowercase letter/s above the bars were not significantly different (*p* < 0.05). * Renewal MI formulation. MCPM-PLS numbers indicate their weight percentage in the powder.

Biofilms formed on all surfaces showed significantly higher WIG compared to WSG. For the WSG, biofilms formed on the PLS-containing formulations contained significantly less glucan compared to the control formula 50% and 62% for 8-4 and 16-8, respectively. The biofilms on the 16-8 formula had significantly less glucan than the 8-4 formula (25%) (*p* = 0.048). For the WIG, the biofilms formed on the 16-8 formula contained significantly less glucan than the control (55%) (*p* = 0.001) and 8-4 (*p* = 0.011) (43%) formulations. Additionally, biofilms formed on the 8-4 formula had less glucan than the biofilms on the control formula (20%), although this was not statistically significant (*p* > 0.05) (Figure 4).

**Figure 5:**
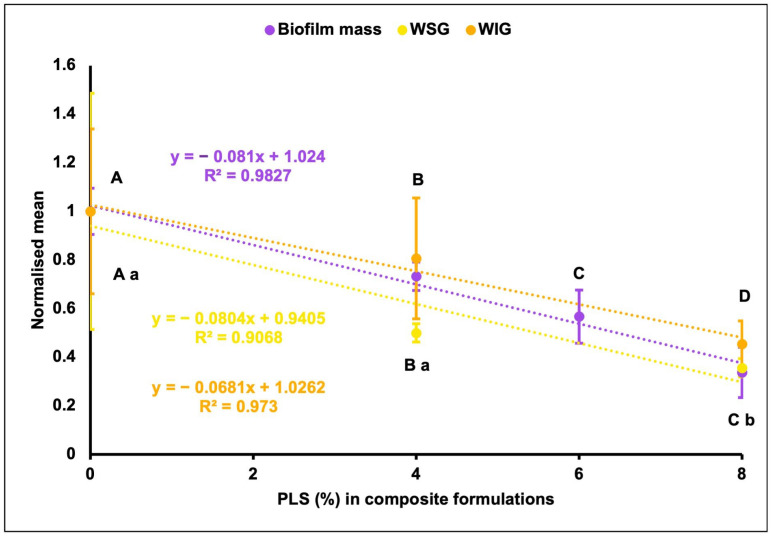
Normalized mean ± (95%CI) biofilm mass, water-soluble (WSG), and water-insoluble (WIG) glucans (n = 3) for different composite formulations. For biofilm mass, the formulations with the same uppercase letters above bars were not significantly different at *p* < 0.05. For WSG and WIG, the formulations with the same uppercase and lowercase letter/s, respectively, below bars were not significantly different at *p* < 0.05. MCPM-PLS numbers indicate their weight percentage in the powder.

When normalizing the biofilm mass, WSG, and WIG data by the control values, linear decreases of up to 66%, 64%, and 55% were noted with the formula containing the highest PLS level (8%). Also, these data showed almost the same linear reduction gradient (≈0.08 ± 0.01) with increasing PLS levels in the formulations (Figure 5).

### 3.5. eDNA Concentration

**Figure 6:**
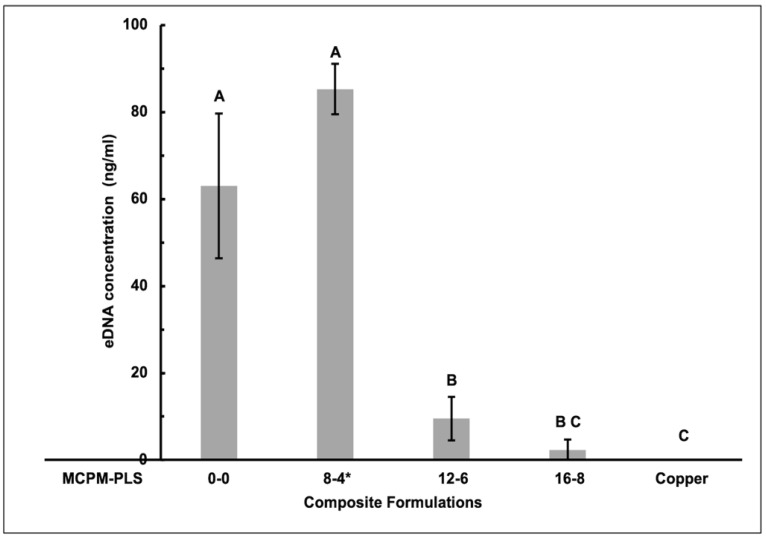
Mean extracellular DNA (eDNA) ± (95%CI, n = 3) (ng/mL) concentration within biofilms formed on the four composite formulations (0-0, 8-4, 12-6, and 16-8) and copper discs (error bars are for three independent repetitions with an average result of three discs per material per repeat (n = 3)). Materials with the same uppercase letter/s above the bars were not significantly different at *p* < 0.05. * Renewal MI formulation. MCPM-PLS numbers indicate their weight percentage in the powder.

The eDNA concentrations in biofilms formed on 12-6 and 16-8 formulations were significantly lower than in biofilms on the control (85% and 92% for 12-6 and 16-8) and 8-4 (88% and 94% for 12-6 and 16-8) formulations. Biofilms formed on the 8-4 formula had more eDNA than the control formula (23%), although this was not statistically significant. Also, biofilms on the 16-8 formula contained a trace amount of eDNA (5%). This was not significantly different from that seen with copper which had no detectable eDNA (Figure 6).

### 3.6. Biaxial Flexural Strength

**Figure 7:**
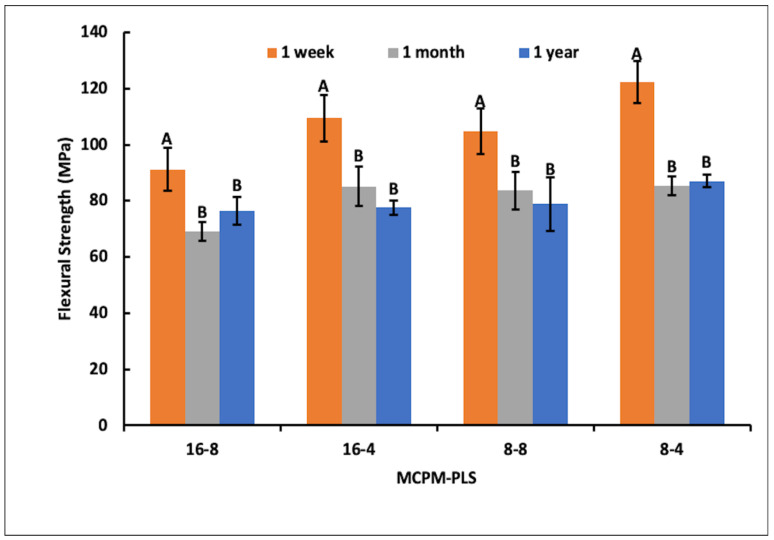
Mean biaxial flexural strengths ± (95%CI, n = 6) (MPa). Materials with the same uppercase letter/s above the bars were not significantly different at *p* < 0.05. 8-4 is Renewal MI formulation. MCPM-PLS numbers indicate their weight percentage in the powder.

Biaxial flexural strength of the four formulations tested were all highest at 1 week and significantly lower (*p* < 0.001) at 1 month compared to 1 week. Results at 1 year, however, were not significantly different (*p* = 1.00) from those at 1 month (Figure 7). 

**Figure 8:**
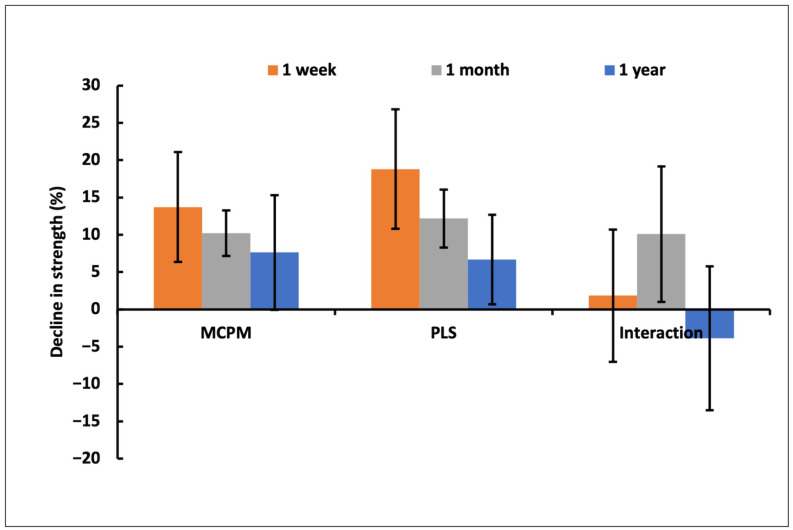
Percentage (%) decrease in strength upon doubling MCPM or PLS concentration and interaction between the 2 variables ± (95%CI, n = 6).

Factorial analysis showed doubling MCPM and PLS caused a 14% and 19% decline in strength at 1 week, respectively. At 1 month, there was a significant interaction effect between MCPM and PLS variables. At 1 year, the effect of these variables was not significant (*p* = 0.05) (Figure 8).

## 4. Discussion

In this study, composites have been produced with re-mineralizing MCPM and antibacterial PLS particles. Together with 4META, these components have previously been shown to aid bonding to enamel and sealing of caries-affected dentine [4,20]. This study aimed to gain a further understanding of how early release of PLS affects biofilm formation. In antibacterial studies, the ratio of MCPM-PLS was fixed at 2 as this gave optimal sealing features in previous work [20]. Additionally, the study looks at how these two components individually affect mechanical properties at different times through varying the MCPM-PLS ratio and the use of factorial design.

For the microbiological studies, trends with increasing the PLS concentration were important, so the PLS/MCPM was fixed. For the mechanical data, the effects of increasing the re-mineralizing MCPM and antibacterial PLS variables, however, were both important so a factorial analysis method was employed. This used two additional (16-4 and 8-8), instead of the 12-6 and 0-0, formulations. Together with the 16-8 and 8-4 formulations, these enabled the determination of the effect of doubling each variable separately. The 0-0 formulation was not included as both variables are simultaneously removed making it impossible to separate the two variable effects.

The above results showed that, apart from solution acidity, all microbiological properties investigated were significantly affected by the level of MCPM-PLS in the composites. Null hypotheses that MCPM-PLS level does not affect biofilm mass, viable colony forming units, WSG, WIG, and eDNA can therefore be rejected. Doubling either MCPM and/or PLS levels significantly decreased the biaxial flexural strength at 1 week. Results at 1 year, however, were not significantly affected by MCPM and/or PLS doubling. Results at 1 month and 1 year were significantly different from those at 1 week but not from each other. Mechanical test null hypotheses can therefore be partially rejected.

CV absorption and glucan levels showed a similar linear decline with an increase in PLS. Conversely, bacterial viability and eDNA were similar for the 0-0 control and 8-4 (Renewal MI) formulations but exhibited an abrupt change between the 8-4 and 16-8 formulations. This may be a consequence of the release of PLS [14] reaching and exceeding a minimum inhibitory concentration with the 12-6 and 16-8 formulations, respectively. Higher PLS concentrations could reduce eDNA levels through a combination of eDNA solubilization [21] and a reduction in bacterial numbers.

Early PLS release into the water from the 0-0, 8-4, 12-6, and 16-8 composite surfaces was previously shown to be proportional to the square root of time and approximately proportional to that in the material [14]. Whilst this plateaued with the 8-4 formulation after 24 h, for the higher PLS content materials, the diffusion-controlled release continued for over 18 weeks. Cationic PLS may form strong ionic interactions with negatively charged groups on glucans, eDNA, and bacteria. As the PLS is highly water-soluble, its early release and interactions with these bacterial components might enhance their dispersion and reduce biofilm attachment to the composite surface. Alternatively, PLS interactions with the proteins or bacteria might reduce production of the biofilm components or have direct bactericidal action for example, by membrane disruption [22].

When diffusion-controlled, the amount of PLS released per hour is inversely proportional to the square root of time and therefore declines over time. A declining release rate could allow the biofilm to develop. If continuing PLS release is trapped inside by ionic interactions, for the 12-6 and 16-8 formulations it could then accumulate at the composite/biofilm interface. The 90% drop in viable bacteria for the 16-8 formulation might then be due to biofilm PLS concentration approaching a critical minimum inhibitory concentration before 48 h. For a composite to be considered antibacterial, however, according to ISO 3990, there should be a 99% kill [3]. This might be achieved in future work by further increasing the PLS content. Alternatively, there might be a greater reduction in viable bacteria on the 12-6 and 16-8 formulations, due to continuing PLS accumulation in the biofilm if studies are conducted for longer. This is unlikely to occur with Renewal MI, however, due to the more restricted PLS release kinetics.

The studies were performed at an early time and under anaerobic conditions to assess if the materials might be effective at reducing biofilm formation by residual bacteria beneath a restoration. In a recent clinical trial, it was shown that Renewal MI composite (8-4 formulation) could be placed directly on disease-affected dentine following hand excavation. The presence of the adhesion-promoting monomer 4META, in addition to the MCPM and PLS, enabled effective sealing of the cavity. This was achieved without the need for separate etchant, primer, or adhesive application [4]. Modern tooth restoration advice advocates the removal of only the soft highly infected tooth structure [23]. Underlying affected dentine, which has lower levels of bacteria, is left in place to re-mineralize. Minimally invasive tooth restoration relies upon effective sealing [24] to reduce nutrient ingress and space for bacterial biofilms to develop. Materials that additionally provide early antibiofilm action, however, could be beneficial if the seal is imperfect.

Shrinkage of composites during polymerization and ineffective bonding can lead to gaps at the tooth restoration margin. Studies have shown that the addition of PLS and MCPM aids composite sealing of disease-affected tooth structures and enables gaps to self-repair through mineral precipitation [5]. Furthermore, these hydrophilic components can encourage water sorption-induced expansion to compensate for polymerization shrinkage. Higher levels of PLS might further enhance these features in addition to reducing biofilm growth. Care must be taken, however, to ensure this does not cause excessive expansion or reduce composite strength [5].

Previous work with the 0-0, 8-4, 12-6, and 16-8 composites [14] showed increasing PLS resulted in a linear decline in *S. mutans* biofilm biomass, as determined by staining with crystal violet, and thickness assessed by confocal microscopy. Cationic CV will bind to negatively charged biofilm components. Of the dry biofilm components, 10-20% is glucan, and ~40% is proteins [25]. The poor correlation between CV absorbance and bacterial counts or eDNA suggests they, in comparison, provide a relatively minor contribution to biomass and CV absorption.

In the new work reported here, the CV absorbance for the 0-0 control has increased by a factor of 1.5 compared with the earlier study [14]. This is likely due to biofilms being grown for 48 h instead of 24 h, with the medium (supplemented with sucrose), renewed after 24 h. Additionally, biomass reduction for the 16-8 compared with the 0-0 control formulation increased from 28% [14] to 66% in this new work. This has enabled higher confidence in the detection of differences between samples. The previous observation of higher dead bacteria percentages with the 12-6 (60%) and 16-8 (80%) formulations provides evidence that the PLS can reach bactericidal concentrations in the biofilms [14]. The 90% reduction in viable bacteria for the 16-8 compared with control formulations in the new work might therefore be due to a combination of both reduction in biofilm thickness and bactericidal action of PLS.

Survival of *S. mutans* in low pH is higher in a biofilm than in a planktonic culture [26]. An acidic environment also enhances acid-base interactions, mediated by eDNA, that promote bacterial aggregation [27]. The lack of change in the acidity of the surrounding medium with composite composition suggests it is not associated with the biofilm mass. This and the pH values are in close agreement with a previous study on growth media around *S. mutans* biofilms [28]. These bacteria are known to lower pH to a level they can tolerate to outcompete other bacteria [29].

The 12-6 sample was not included in the glucan test as results with the other samples were sufficient to demonstrate linear decline with increasing PLS concentration. Exopolysaccharide content, assessed using the 0-0, 8-4, and 16-8 formulations, was sufficient to demonstrate a linear decline with increasing PLS content. Additionally, normalized exopolysaccharide data were comparable with those obtained through normalized CV staining results. Analysis of the 12-6 data was therefore not undertaken for WIG and WSG studies. The WSG and WIG samples were freeze-dried and resuspended in a reduced volume to increase their concentration above the lower limit of sensitivity of the phenol-sulfuric acid microassay. Alternatively, a lower volume of PBS could have been used to solubilize the glucan, but this risked poor solubilization of the biofilm components.

Water-soluble (WSG) and water-insoluble (WIG) glucans are synthesized by *S. mutans* glucosyltransferases (Gtfs) from sucrose, mainly to promote bacterial adhesion and colonization [8]. The acidic environment inside biofilms, created by acidogenic organisms, stimulates glucan production and biofilm growth. Glucan may also provide binding sites for more acidic organisms to join the low-pH environment [30,31]. WIG being significantly higher than WSG agrees with a previous study [32]. Correlation between exopolysaccharide mass and CV absorbance is consistent with it, and the Gtfs proteins that produce it, are a major component of the biofilms.

Several studies have assessed the eDNA in biofilms with dsDNA dyes and fluorimetry [33,34]. The Qubit fluorometer showed no evidence of eDNA in biofilms formed on the copper. This is consistent with a trace amount of biofilm and indicates an eDNA concentration below the fluorimeter limit (<10 pg/µL).

Some studies have proposed the source of eDNA is cell death and autolysis as the source, while others suggest eDNA is actively secreted from bacteria [35]. Biomass reduction in mature biofilms upon DNase addition suggests that eDNA affects biofilm stability, structural integrity, and mass formation [35,36,37]. eDNA also promotes *S. mutans* and glucan-mediated biofilm adherence to surfaces [35,36]. Imaging has shown the eDNA can be connected to the substratum and to near, as well as distant, bacterial cells by a highly structured network of nanofibers [36]. eDNA may therefore affect surface and intercellular adhesion as well as the stability and strength of the ECM. Moreover, eDNA may act as a reservoir for horizontal gene transfer and be a nutrient source for bacterial cells because of its richness in carbon, nitrogen, and phosphorous [35].

Previous studies concluded that eDNA can interact with glucan in *S. mutans* biofilms, but as seen in this work, the amount of eDNA does not depend on the amount of glucan in the biofilms [28,38,39]. Instead, the correlation between eDNA and viable bacteria aligns with the proposed hypothesis that eDNA is naturally secreted from viable rather than dead bacterial cells [28].

Weakening biofilm stability by reducing the amount of exo-polysaccharide and eDNA or the number of viable bacteria is likely to decrease the biofilm’s pathogenicity and allow for the reparative process (re-mineralization) to efficiently take place. Moreover, increasing the PLS level leads to a higher percentage of the PLS in the composite being released [14]. In the clinical situation, an accumulation of PLS under the restoration and in the dentinal tubules might occur from the formulations with higher than 6% PLS thereby providing antibacterial action. Renewal MI may also reduce early biofilm binding to the composite via electrostatic interactions and solubilizing of biofilm components by released PLS.

The above studies have confirmed the potential antibacterial benefits of adding more PLS into the Renewal MI formulation, but it can also reduce early strength. ISO 4049 suggests mechanical testing at 24 h with conventional composites [15]. For the new materials in this investigation, however, both PLS and ions for re-mineralization are released which can reduce mechanical properties. Longer-term strengths and proving they reach a stable level were, therefore, more important than results at 24 h. Polymerization of dental composites can continue after the light is turned off. Curing both sides for 40 s then leaving for 1 day for both the microbiological and mechanical studies ensured the polymer matrix phase was fully cured, stable, and reproducible before any liquid immersion. The decline in biaxial flexural strength at 1 week with increasing PLS and MCPM may be due to a lack of silane coupling agent on these particles. This may have been partially addressed through the addition of 4META in the monomer phase [40].

The decline between 1 week and 1 month of placement in water is likely due to a combination of component release and water sorption. These processes reaching equilibrium at 4 weeks would be consistent with mechanical properties changing little between 4 weeks and 1 year. Water sorption could reduce strength by plasticizing the matrix or possibly by hydrolyzing the silane between the matrix and glass filler particles. The lesser reduction in strength with doubling MCPM and/or PLS at later times might be a consequence of the MCPM’s ability to react with absorbed water. As other calcium phosphate compounds of lower density can be formed, this provides a mechanism to fill cracks and defects. With higher MCPM and PLS, the lower strengths may limit application to lower load-bearing applications or to placement beneath other composites.

Regarding the limitations, the experiments’ model did not include teeth substrate, which may affect the biofilm formation systems and the PLS released from the composites. Also, the results were obtained from short-term aged mono species (*S. mutans*) biofilms. Probably a longer-term study of multispecies biofilms would give a different result. A previous study found that UA159 *S. mutans* biofilm has less exo-polysaccharide and eDNA than multispecies biofilms [41]. This may considerably affect the treatment outcome, whereas multispecies biofilms could be less susceptible to antimicrobial agents than mono-species biofilms [42,43]. An additional limitation is that the current quantified biofilm components give no information about the biofilms’ architecture or the effect on the biofilm dimensions. The absence of such variables might under- or overestimate the antibacterial effect of composites.

Further work is now required to assess how the additives, or their release, affect paste stability and long-term properties. These include water sorption, mass loss, surface porosity, cavity sealing capability with sound versus caries-affected tooth structures, wear resistance, bond strengths, fatigue, aesthetics, and color stability. Additionally, characterization of the fracture surfaces using SEM and Raman mapping could provide a better understanding of mechanical properties.

## 5. Conclusions

Increasing PLS levels in composite formulations causes a linear reduction in biomass and exo-polysaccharide. An abrupt reduction in eDNA and viable bacteria in surface biofilms is observed when the level of MCPM and PLS in Renewal MI is doubled. Composite strengths following 1 week of water immersion are affected more by doubling MCPM and PLS levels than those after 1 month and 1 year.

## 6. Patents

This work is under the following licensed patents: Composites formulations with reactive fillers (US8252851 B2, EP2066703 B1, US20100069469, WO2008037991 A1) and material formulations with cationic polymers (PCT/GB2014/052349, WO2015015212 A1, EP3027164 A1, Us20160184190).

## Data Availability

The datasets used during the current study are available from the corresponding author upon reasonable request.
